# Optical Trajectory Manipulations Using the Self-Written Waveguide Technique

**DOI:** 10.3390/polym12071438

**Published:** 2020-06-27

**Authors:** Ra’ed Malallah, Derek Cassidy, Min Wan, Inbarasan Muniraj, John J. Healy, John T. Sheridan

**Affiliations:** 1 School of Electrical and Electronic Engineering, College of Engineering and Architecture, University College Dublin, D 4 Dublin, Ireland; derek.cassidy@ucdconnect.ie (D.C.); min.wan@ucdconnect.ie (M.W.); inbarasan.muniraj@ucdconnect.ie (I.M.); john.healy@ucd.ie (J.J.H.); 2 Physics Department, Faculty of Science, University of Basrah, Garmat Ali, Basrah 61004, Iraq

**Keywords:** self-written waveguide, photo-polymer, fiber optics

## Abstract

This study is novel for several reasons: We used a thin drop cast layer of dry photosensitive materials to study the behaviors of wet photopolymer media using microscopic distances during the Self-Written Waveguide (SWW) process; then, we examined the self-trajectories formed inside the solid material. The results provide a framework for theoretical and experimental examinations by handling the effects of manipulating the alignment of fibers. The other main advantage of these techniques is their lightweight, easy to process, highly flexible, and ultimately low-cost nature. First, the SWW process in wet photopolymer media (liquid solutions) was examined under three cases: single-, counter-, and co-fiber exposure. Then, the SWWs formed inside the solid material were examined along with the effects of manipulating the alignment of the fibers. In all cases, high precision measurements were used to position the fiber optic cables (FOCs) before exposure using a microscope. The self-writing process was indirectly monitored by observing (imaging) the light emerging from the side of the material sample during SWW formation. In this way, we examined the optical waveguide trajectories formed in Acrylamide/Polyvinyl Alcohol (AA/PVA), a photopolymer material (sensitized at 532 nm). First, the transmission of light by this material is characterized. Then, the bending and merging of the waveguides that occur are investigated. The predictions of our model are shown to qualitatively agree with the observed trajectories. The largest index changes taking place at any time during exposure, i.e., during SWW formation, are shown to take place at the positions where the largest exposure light intensity is present. Typically, such maxima exist close to the input face. The first maximum is referred to as the location of the *Primary Eye*. Other local maxima also appear further along the SWW and are referred to as *Secondary Eyes*, i.e., eyes deeper within the material.

## 1. Introduction

Many different types of self-process photosensitive materials (photopolymers) have been studied for producing Self-Written Waveguides (SWWs) [1,2,3]. The greatest advantage of such materials is their ability to obtain a large stable index change during exposure. Sensitivity to light occurs quickly, which is typically reproduced during the photo polymerization process. These characteristics facilitate the investigation of self-writing by producing consistent experimental results and making them suitable for device applications [4,5,6]. Other advantages include their optical quality (a relatively low scatter), low cost, good spatial frequency response and, for volume ratings, high diffraction efficiency [7,8]. 

That self-written structures can form in a photopolymer was first established by Kewitsch et al. [9]. However, it was observed that the material response has non-ideal characteristics. Such material effects, such as dye absorption, constrain photopolymer material development and applications [10,11,12]. To maximize the potential of these materials, deeper insights into the photo–physical and photo–chemical evolution taking place during photo-polymerization (throughout the material’s volume over time) are necessary. Studies have examined the use of SWWs in photopolymers to couple (including repairing by self-healing) between optical fibers, to form micro-tips at the ends of optical fibers, to create biologically inspired microstructures, and to implement strain sensors [2,7,13,14,15]. Photopolymer materials provide all of these applications, which require high-contrast refractive-index changes and high recording sensitivity [7]. It should also to be noted, however, that many recent studies into self-guided (self-trapped) optical beams have involved beams propagating in slab waveguides or nonlinear bulk media. When a light beam propagates inside a photopolymer material, the diffraction can be compensated as the refractive index increases [16]. Ideally, a highly photosensitive material that generates stable long-lasting refractive-index changes in response to illumination at a specific wavelength is needed [17,18].

The work presented here is organized as follows: First, wet photosensitive materials (liquid solutions) that have been previously examined for SWW formations are introduced. In this section, recent studies on using wet photosensitive materials are outlined. These studies feature several applications that focus on applying liquid materials (e.g., the optical gel used for splicing optical fibers). In this section, we give readers a good overview about the best methods for our material and why (i.e., whether it is a liquid or solid). The advantages of using dry materials are also explained. This section includes a discussion on self-writing mechanisms and an investigation into the nonlinearity of our photosensitive material. The novelty of using a thin drop cast layer of dry photosensitive materials (using microscopic distances) is studied intensively. The experimental set-up and material preparation procedures are presented. Then, several waveguiding structures are fabricated. Finally, the model used for the simulations is described, and a comparison of the measured and predicted results is carried out. This technique can also be considered self-induced waveguides that are fabricated by propagating light trajectories through photopolymer media [19,20].

## 2. Self-Written Waveguide Evolution

The self-writing process is indirectly monitored by observing the transmitted light distribution emerging from the output of a sample during its exposure. Light distribution is also examined by capturing images of the light scattered from the sample side (along the waveguide path). In our experiments, the output beam is initially observed to become narrow and more intense during exposure, as the index within the material increases with the formation of the SWW [18,19,20,21]. A 3D model (combining material and electromagnetic effects) is necessary to describe the creation of SWWs as the exposed light beam distribution in *x* and *y* propagates through a photopolymer material [22,23,24,25,26,27,28].

The phenomenological model developed in this work can be used to predict both the evolution of light intensity distribution and channel formation inside the material during exposure [19,28,29,30,31,32,33]. The results obtained using the improved model provide a more detailed description of waveguide evolution. Then, the predictions for several characteristics in the modified model are observed inside the material during the self-writing process. The behaviors of both the wet and dry samples during the SWW process are then examined. This work is performed on relatively thin drop cast layers over small propagation distances (microscopic distances) with an exposing power of *P*_0_ = 0.1 mW (i.e., each fiber has *P*_0_ = 0.1 mW). The simplest case for self-writing the exposed beam profile is then examined. All cases studied involve the use of a Gaussian mode as the writing beam, i.e., the writing beams are input using single mode optical fibers. The material preparations for the cases examined here are described in detail in [34].

**Figure 1:**
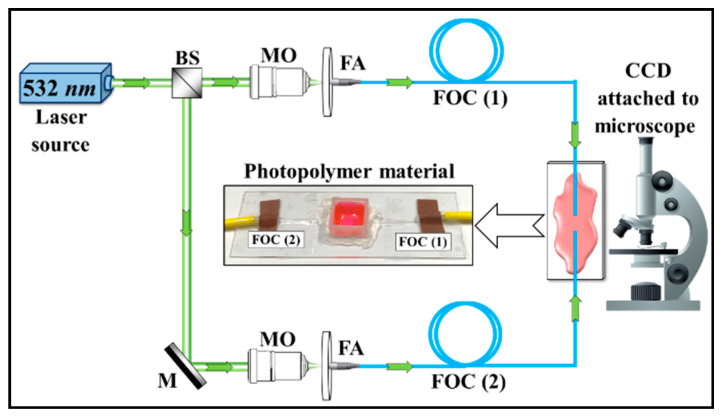
Optical setup for observing self-written waveguide formation through a photopolymer material to observe the dual single-mode fiber optic beam exposure.

The experimental system used is illustrated in Figure 1, with an actual setup inset. A beam splitter (BS) is used to divide the green laser beam (532 nm) into two beams. Then, each beam is coupled into a single-mode (SM) Fiber Optic Cable (FOC) type (P1-460B-FC-5) using Microscopic Objective lenses (MOs) (Olympus/RMS4X) and Fiber Adapters (FAs) (Qioptiq/G067054000). In the experiments, the two fibers were attached to a microscopic slide glass with their ends aligned (using a Luminos, Optical Fiber Alignment Stage) pointing toward each other (each fiber produces a Gaussian beam with *P*_0_ = 0.1 mW). The photopolymer material was drop cast between the ends of the optical fibers to fill the space between them (see Figure 1). The process of SWW formation was monitored using a microscope (BRESSER Biolux NV 20x-1280x). Initially, the goal was to observe whether the medium exhibited the ability to interconnect the fibers and, therefore, possibly other integrated optical devices, as well. Silica based photopolymer materials were used to combine the integrated optical devices [35]. Typically, the core size of a formed waveguide is around ~9 µm, with an index difference between the core and a cladding of ~10^−3^. The Numerical Aperture (NA) of such a single mode fiber will typically be around 0.05 ≤ NA ≤ 0.15. The minimum bending radius of the waveguide fundamentally depends on the core size [36].

In this work, the photopolymer material used consists of several components: Polyvinyl Alcohol-PVA (binder), Acrylamide-AA (monomer), Bisacrylamide-BA (cross-linker), and Triethanolamine-TEA (electron donor) [13]. The dye used here is Eosin–Yellowish (EY) (C_20_H_6_Br_4_Na_2_O_5_), which is photo-sensitive to green light at a wavelength of λ = 532 nm and acts to initiate the polymerization process [12,21,37]. A Gaussian beam at this wavelength emerges from the SM fiber inside the photosensitive material (see Figure 1). The light then propagates into the photosensitive material. The resulting refractive index changes generated due to polymerization make the SWW index higher than that of the surrounding material. Self-focusing will then take place [38,39]. Stable SWWs are formed if the exposing wavelength induces long lasting refractive index changes.

## 3. Results and Discussion

To predict waveguide formation and, therefore, be able to optimize fabrication, the evolution of the refractive index distribution needs to be experimentally monitored. However, during self-writing experiments, there is no simple method to directly measure the refractive index distribution induced in *x*, *y,* and *z* over time without destroying the sample. Therefore, we indirectly observed waveguide formation by measuring the transmitted and scattered light.

In this paper, the self-writing process is examined in two phases (liquid and solid) of the same material. First, the behaviours of SWWs inside the liquid photopolymer material are studied for three cases: single-propagating beam, counter-propagating beam, and co-propagating beam exposures. Second, the SWWs are formed inside the solid material layers (as above), which involve the misalignment of fibers. In all cases, the FOCs are positioned with microscopic accuracy. The self-writing process is indirectly monitored by observing the light emerging from the sides of the material sample during exposure. As the SWW forms, this imaged light narrows and becomes more intense as the index within the material increases in response to the waveguide formation. Numerical simulations of the self-writing process are then performed and compared to the observed results.

### 3.1. Liquid Photopolymer

**Figure 2:**
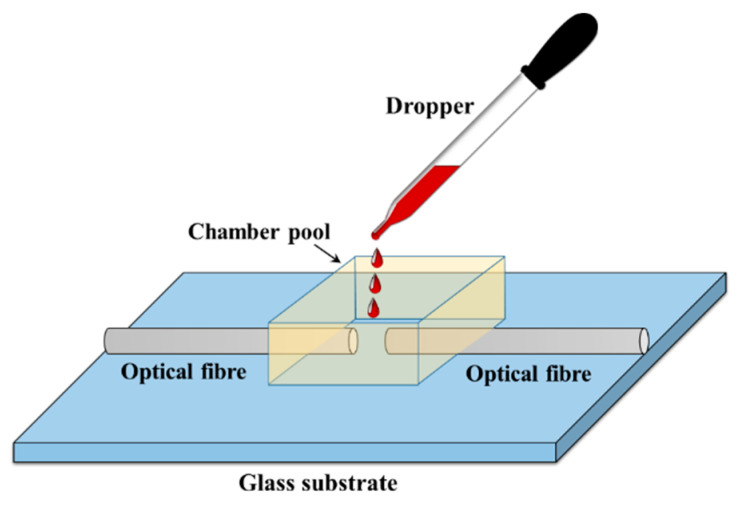
Drop cast photopolymer solution onto the gap between the optical fiber ends.

First, the liquid photopolymer material is examined. The photopolymer material preparation is discussed in [34]. The material’s composition and preparation are similar to those used for holography. Single-mode optical fibers, with core diameters of 9 µm (125 µm for the cladding), were used in the examined configurations. The optical fibers were placed on a microscope slide (25 mm × 75 mm) using an optical fiber linear translation stage (Luminos, optical fiber alignment stage). The photopolymer material was then drop cast over the two optical fibers (or one optical fiber), as illustrated in Figure 2. A small shallow chamber pool was formed around the gaps between the fibers (the walls of this pool were made by cutting 2–4 mm off the top of a cuvette) to ensure they were covered by sufficient photopolymer material. A green laser light (532 nm, 0.1 mW) was then used to expose the material. The optical fiber cables were held in place by tape attached to the microscopic glass substrate (in the particular arrangement used for each study). Then, the photopolymer solution was drop cast to cover the fiber cable under dark room conditions. All experimental observations were captured using a microscope.

**Figure 3:**
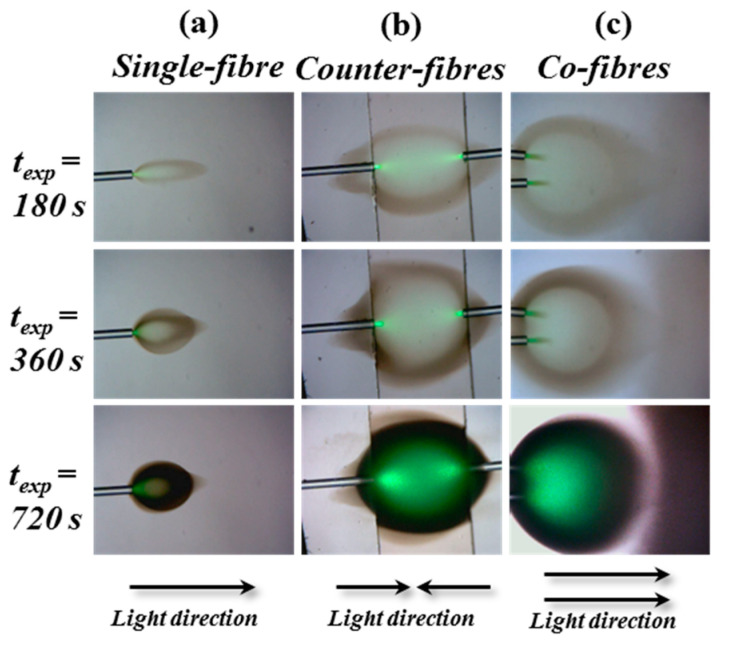
Microscope images of the spherical shapes created at the end of an optical fiber (*λ* = 532 nm and *P*_0_ = 0.1 mW) inside the liquid photosensitive solution. The three exposure geometries involve illumination using (**a**) single-fiber, (**b**) counter-fibers, and (**c**) co-fibers. In each case, the results for three exposure times (180 s, 360 s, and 720 s) are presented.

In this situation, exposure first acts to reduce the amount of inhibitor oxygen, and eventually polymerization takes place. The free radical chains generated eventually terminate. Ultimately, a dense polymer material with a higher refractive index is formed. An irregular pattern is thus formed, which is observed to be discolored and have a spherical shape (see Figure 3).

In Figure 3, different variations of the exposure setup are used to examine the results for different types of illumination. In all cases, spherical shaped staining within the photopolymer solution was observed. The three sets of results for the three arrangements of optical fibers examined show the evolution of similar spherical staining at the same three exposure time intervals: 180 s, 360 s, and 720 s. The measured transmission through the solution is not like that found during the formation of SWWs. Even though the refractive index changes, it does not appear to change in a way that produces guiding. Instead, the light is scattered, and the material acts to diffuse it, effectively obstructing the propagation of the light. The use of liquid solutions does not appear to provide a practical way to produce SWWs.

### 3.2. Solid Photopolymer

**Figure 4:**
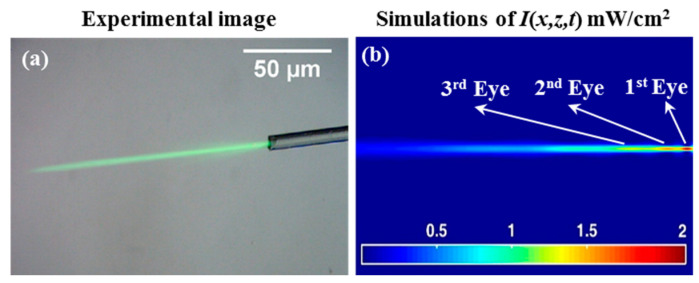
Light incident (from right to left) from a single mode optical fiber, *t_exp_* = 1600 s of *λ* = 532 nm and *P*_0_ = 0.1 mW: (**a**) experimental image results and (**b**) the corresponding numerical intensity predictions, *I*(*x,z,t*) mW/cm^2^.

Solid photopolymer media were examined. In these cases, the drop cast material (see Figure 2) was allowed to dry to form a solid layer before exposure. Figure 4a shows the experimental results of an attempt to create self-written optical waveguides using the illumination from single mode fiber optics (Gaussian intensity profile, *P*_0_ = 0.1 mW). Here, essentially the same set-up as that shown in Figure 1 was used. The light beam was observed to expand (diverge) as it propagates in the solid homogenous medium. Eventually, the light begins to focus to create a narrow channel that changes the beam intensity shape, confirming the generation of an SWW (see Figure 4a). Moreover, the optical waveguide channel along the direction of propagation inside the sample is caused by the self-focused and self-trapped process [3,7,21,40,41]. These results were compared to the corresponding numerical simulations using the MATLAB R2015a software, as shown in Figure 4b. The predictions in Figure 4b show the light intensity distribution within the material at *t_exp_* = 1600 s. The material here undergoes a physical change due to the propagating light becoming absorbed by the photosensitive material.

One effect identified during previous studies is the appearance of locations with high intensity during SWW formation. At time *t*_0_, early in the exposure, peak intensity is observed close to the face of the optical fiber, where light is input into the material. This peak moves along the propagation axis at *t_exp_* > *t_0_*. Figure 4b shows the *Primary Eye* (1st *Eye*), which refers to the region with relatively higher intensity [42]. The presence of the *Primary Eye* at a location within the material indicates that the diffraction (spreading) of the propagating light is beginning to be overcome by an increase in the refractive index along the waveguide path. After initially forming, the *Primary Eye* is observed to move forwards along the propagation axis. However, later it is seen to move back towards the input face. As the exposure continues, the index continues to evolve along the propagation path [32,42].

As the refractive index increases, the SWW forms a cross-sectional refractive index (in the case of Gaussian beam illumination) that increases along the waveguide trajectory. Depending on the exposure intensity and the dye and monomer available, the refractive index increases by different amounts at different places. Due to these increases, new intensity maxima (local focus points) appear and are referred to as *Secondary Eyes* (the 2nd and 3rd Eye). This growth in the waveguide profile directly leads to the existence of the *Secondary Eyes* and their movement closer together and towards the input over time. The secondary eye appears to the right of the primary eye of intensity (further away from the input) [32]. In the numerical simulation results presented in Figure 4b, the secondary eyes just begins to appear. It is also less localized (more spread out) along the SWW (in *z*) than the *Primary Eye*. To the left of the second eye (2nd), a third *eye* (3rd) eventually appears. The time and location at which the *Primary Eye* and *Secondary Eyes* form depends on the writing beam (intensity) and the material, e.g., the initial refractive index of the material and the maximum (saturation) index change that can be induced within the material [42,43]. As the refractive index profile continues to increase, the waveguide will, during exposure, act more strongly on the light. This will lead to the appearance of more eyes, until, eventually, polymerization ends due to the lack of a monomer. This model predicts a maximum percentage normalised transmission of light for propagation over a distance of 80 µm, i.e.,$\;(I_{out}/I_{in})\times 100\%\;$= 49.65%.

**Figure 5:**
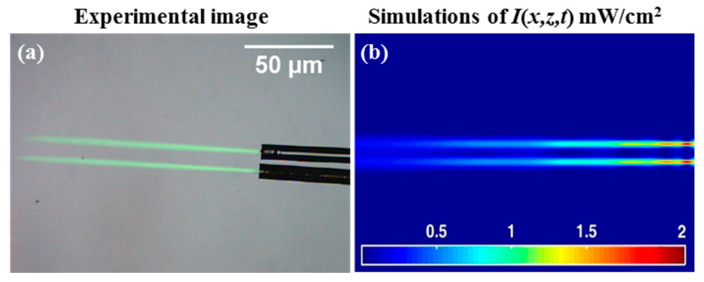
(**a**) Experimental observations (from right to left) for the SWW formed by exposure, *t_exp_* = 1600 s of *λ* = 532 nm and *P*_0_ = 0.1 mW (i.e., each fiber has *P*_0_ = 0.1 mW); two parallel single mode fiber optical cables; (**b**) simulated light intensity distributions, *I*(*x,z,t*) mW/cm^2^.

Next, the formation of SWWs is examined under exposure using two parallel optical fibers (co-fibers), separated by ~9.13 µm, which simultaneously expose the AA/PVA with a power intensity *P*_0_ = 0.1 mW (i.e., each fiber has *P*_0_ = 0.1 mW) at *λ* = 532 nm (see Figure 5a). The corresponding predictions of the theoretical model are presented in Figure 5b. In the numerical predictions, good qualitative agreement is observed with the experimental results. This suggests that our model reasonably accurately describes the dynamic photochemical and optical processes taking place within the material over the time period examined.

The experimental image was generated along the two waveguide trajectories (path) within the sample. The image contains light scattered out from the material, but at least some light is absorbed during expose and then re-emitted. The subtle features of the exposure’s light pattern during the self-writing process was imaged, including observations on the appearance of the *Primary* and *Secondary Eyes* that were predicted by the model [42]. The two waveguides formed in this case follow separate paths during the self-writing process (shown when *t_exp_* = 1600 s). These paths are parallel, exhibiting no convergence, divergence, or bending of their trajectories. The theoretical predicted value of the maximum percentage normalized transmission for both beams is identical to 49.65% for propagation distances of 80 µm (for each beam), the same as the results for the single beam case. Therefore, the two trajectories do not appear to interact and are independent of one another. Such an arrangement of writing fibers can be used for many potential applications [2,7,13,14,15].

**Figure 6:**
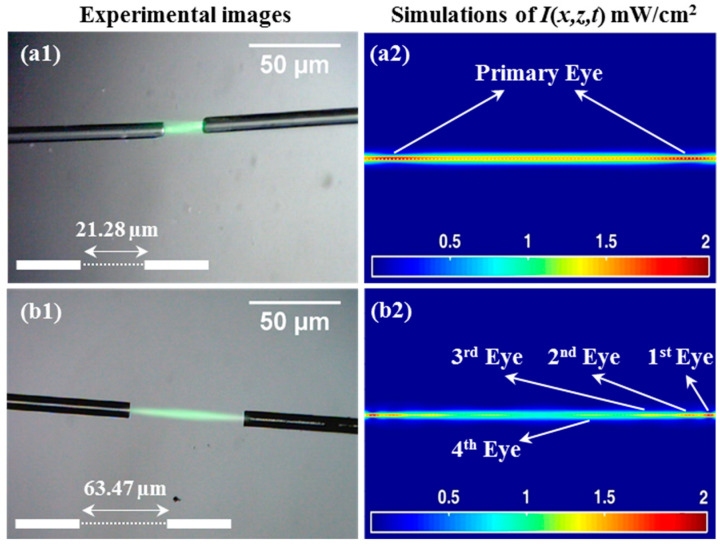
SWW for coupling using counter-propagation beams from single mode optical fibers. *t_exp_* = 1600 s of *λ* = 532 nm and *P*_0_ = 0.1 mW (i.e., each fiber has *P*_0_ = 0.1 mW). The FOCs are aligned and separated by (**a1,a2**) 21.28 µm and (**b1,b2**) 63.47 µm.

To further investigate the processes of self-writing, the outputs of two single mode optical fibers were next carefully aligned and arranged facing each other (see Figure 6). Again, the FOCs were placed onto a glass plate, held in place by tape, and a solution of photopolymers was drop cast onto them under dark room conditions. The photopolymer solution covering the fibers was then left to dry for 24 h.

The two optical fibers were separated by gaps of 21.28 µm (Figure 6(a1,a2)) and 63.47 µm (Figure 6(b1,b2)). All the measurement values listed here (i.e., distances and angles) were calculated from image data using the ImageJ software package [44]. Two beams then exposed the AA/PVA simultaneously from the optical fibers. The beam then counter-propagated (in opposite directions) directly towards the opposite fiber. The resulting passive connection would operate equally well for beams travelling in either direction (reciprocity). The exposure process continued for 1600 s (each fiber has *P*_0_ = 0.1 mW), thus allowing the SWW process to complete (to saturation). After this time, no further evolution of the channel was observed.

The corresponding simulation results are presented in Figure 6(a2,b2). A good qualitative agreement with the experimental results was observed. This technique for connecting two optical fibers is potentially very advantageous, as light from the actual fibers “automatically” generates the optical waveguide channel. In the simulation, the movement of the symmetrically located eyes (from both the left and right illuminations) can be seen along the propagation axis.

As noted, the experiments were performed with the fibers placed (a) 21.28 µm apart and (b) 63.47 µm apart. This enabled us to examine the effects of the separation distance on the process by comparing the results (see Figure 6(a2,b2)). In both cases, simultaneous coherent exposures clearly produced waveguides. Our model also produces qualitatively consistent predictions. The model predicted the increase in optical power along the waveguide as the two exposing beams meet and then cross. This can be seen in Figure 6(b2), where the optical power level is identified by the colour. Yellow indicates the highest intensity value that occurs in the middle of the gap. In Figure 6(a2), an increase in optical power was also seen as the two beams cross.

In Figure 6(a2), the two symmetrically located *Primary Eyes* are shown to appear in the region in front of both the optical fibers. In Figure 6(b2), the *Primary Eye* (1st) and a series of higher order eyes (2nd, 3rd, and 4th) are positioned symmetrically along the SWW [40]. This indicates that the two counter-propagating beams are present and confined. The *Secondary Eyes* can be seen in Figure 6(a2), but due to the short distance between the two fibers, the intensity is more uniform across the gap. However, as the gap between the two fibers increases (see Figure 6(b2)), the *Secondary Eyes* can be more clearly distinguished from one another as they are more widely separated. Using this model, it is possible to predict the maximum percentage normalised transmission (the value for propagation to the mid-point), if $I_{in\left(1\right)}$ is the input intensity from the right, and $I_{in\left(2\right)}$ is the corresponding intensity from the left. Then, dividing the predicted central intensity,$\;I_{mid}$, by the sum of the two input beams ($I_{in\left(1\right)}=I_{in\left(2\right)}$) gives the fraction of light from each beam propagating that distance, i.e.,$\;\{I_{mid}/\left(I_{in\left(1\right)}+I_{in\left(2\right)}\right)\}\times 100\%\;$= 41.42% at *z* = 10.64 µm (see Figure 6(a2)) and 33.73% at *z* = 31.73 µm (see Figure 6(b2)).

**Figure 7:**
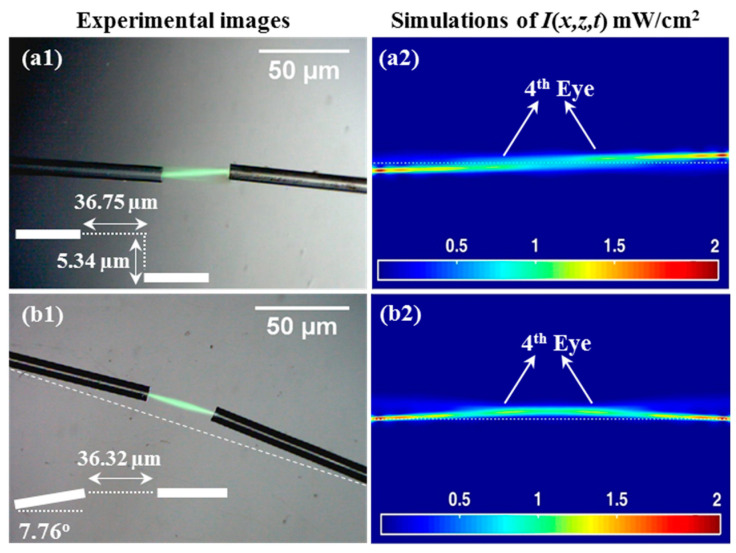
SWW for coupling single mode optical fibers at *t_exp_* = 1600 s of *λ* = 532 nm and *P*_0_ = 0.1 mW (i.e., each fiber has *P*_0_ = 0.1 mW). Experiments and simulations: (**a1,a2**) separated by 36.75 µm with shafting of 5.34 µm and (**b1,b2**) separated by 36.32 µm with an angle of 7.76°.

In Figure 7, we examine cases in which the input optical fibers were not aligned. In this way, the ability of the photopolymer material to help direct the waveguide formation trajectories to produce a waveguide capable of connecting mis-oriented optical fiber cables is demonstrated. In Figure 7(a1,a2), two optical fibers separated by ~36.75 µm with a lateral displacement (or shafting) of ~5.34 µm are examined. In the second case, the optical fiber cables are separated by a distance of ~36.32 µm (Figure 7(b1,b2)). However, in this case, they were not displaced but instead angled with respect to one another, possessing an angular difference of ~7.76°. Again, two identical light beams are simultaneously transmitted along the optical fibers and then propagate inside the drop cast photopolymer material layer. The fibers are oriented so that the beams do not exactly cross propagate. However, they are theoretically predicted to produce a self-written waveguide (see Figure 6). The two beams clearly interacted with each other during the process of SWW formation. The result is a single waveguide rather than two separate waveguides. As an effect, the two waveguides converge, i.e., the trajectories bend toward one another. In this way, a method to connect two separated and misaligned optical fibers was demonstrated.

The experimental results are presented in Figure 7(a1,b1), and the corresponding predictions of our model are shown in Figure 7(a2,b2). There is good qualitative agreement between the two. This model correctly predicts the emergence of the *Primary Eye* and the *Secondary Eyes*. The symmetrical appearance of the eyes in both cases indicates that the two beams are involved in creating the connecting waveguides. The slight bending of the SWW trajectories, which can be seen in Figure 7, indicates the path followed by the light within the formed waveguides. Clearly, a connection is formed between the two optical fiber cables. Consequently, the difficult opto–mechanical alignment currently needed for conventional fusion splicing or mechanical-splicing techniques becomes less critical. Numerically, the model predicts the value of the maximum percentage normalised transmission (in the middle distance between the two beams), i.e., to be 22.63% at *z* = 18.37 µm (see Figure 7(a2)) and 28.42% at *z* = 18.16 µm (see Figure 7(b2)).

**Figure 8:**
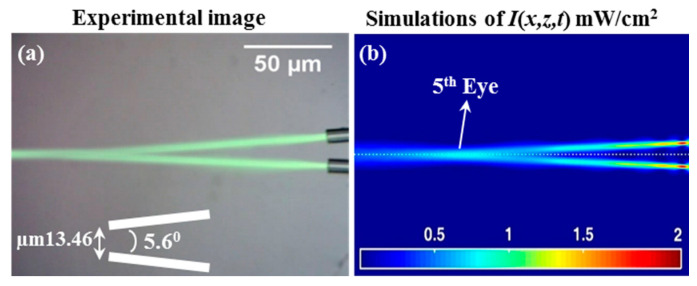
(**a**) Experimental observation image and (**b**) simulated light beam intensity, *I*(*x,z,t*) mW/cm^2^. Self-bending SWW behaviors for *t_exp_* = 1600 s of *λ* = 532 nm and *P*_0_ = 0.1 mW (i.e., each fiber has *P*_0_ = 0.1 mW). Two fibers initially separated by 13.46 μm and an angle of 5.6°. The formation of a *Y*-coupler (*Y*-waveguide). In all cases, the beams travel from right to left.

Figure 8 shows a situation in which two optical fibres are positioned side by side separated by a distance of ~13.46 µm misalignment inwards at an angle of ~5.6°. The resulting waveguides formed within the photopolymer material were then examined, and experiments and simulations were performed. The theoretical model also predicts (in agreement with the experiment results) that a combination of the two waveguides into one will take place; even though the 4th and 5th Eyes have lower values than the first eyes, the 4th and 5th Eyes are visible and clearly exist (see Figure 8b). The predicted existence of a 5th Eye supports the claim that the two optical trajectories combined as the total exposing optical power from both fibres was predicted to combine. To perform the experiment, the two slightly misaligned fibres (relatively tilted by ~5.6°) were attached (by tape) to the microscopic slide. The photopolymer solution was then drop cast. The two beam exposures subsequently took place. The 5th Eye that was predicted to occur following the convergence of the two channels is highlighted in Figure 8b. A *Y*-shaped junction was then created, which is analogous to the *Y*-coupler cables used in optical networks. In this experiment, the beams themselves generated the *Y*-combiner action. The optical axes of two beams intersected inside the photopolymer media such that the two waveguides growing simultaneously from the two beam spots collided with each other inside the photosensitive material. These actions generated by the waveguide process are due to the higher refractive index changing along the direction of the light propagation. This type of non-linear material response can produce a channel with a non-ideal shape. Due to the consumption of dye in the area between the two trajectories, this channel becomes stronger in the coupling light beams (see Figure 8). This leads to even greater interactions between the two channels as the exposure time increases [32,33].

**Figure 9:**
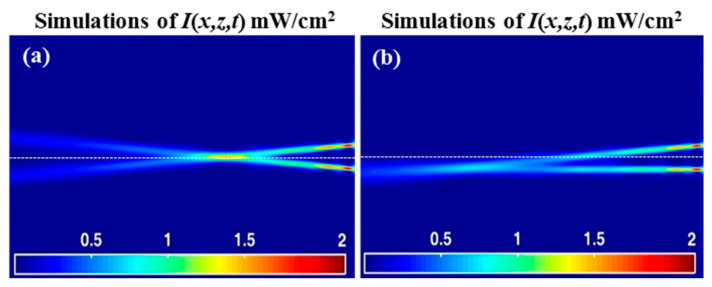
Predicted SWW structures, *I*(*x,z,t*) mW/cm^2^, formed at *t_exp_* = 1600 s of *λ* = 532 nm and *P*_0_ = 0.1 mW (i.e., each fiber has *P*_0_ = 0.1 mW) for (**a**) the *X*-waveguide (> 9°) and (**b**) the off-axis *Y*-waveguide (< 9°).

Based on our experimental results and our simulation software predictions, we found that fibres separated by less than 9° will always combine into a single channel. However, these beams will always cross each other when separated by more than 9° (see Figure 9a) [18,45]. In Figure 9, the predicted SWW structures produced for another example, featuring two beam inputs with different angular separations, are presented. By increasing the angular separation to more than 9°, *X*-coupling structure is formed (see Figure 9a). Figure 9b shows the structures predicted when the upper beam angle is greater than that of the lower beam, but the angles between them are less than 9°. This result is consistent with the results presented in Figure 8; moreover, a *Y*-coupler is formed. Such waveguide structures have many potential applications [2,7,13,14,15,18,45].

## 4. Conclusions

In this research, the ability to fabricate self-written waveguides in a photopolymer material was studied. Poor results were observed when recording in liquid solutions. However, we demonstrated a possibility permanent structure in the solid photopolymer material. In this manuscript, the photopolymer material used is sensitive at 532 nm and was manufactured under room temperature conditions. The novel application of thin drop cast AA/PVA layers during SWW formatting was also successfully demonstrated. The bending of light trajectories during self-written waveguide formation was shown and could be used to form a wide range of potentially useful photonic circuits. To understand the self-bending phenomenon, different input beam (exposure) arrangements were compared experimentally and numerically.

In this paper, we first examined the use of a photopolymer solution (liquid) as an SWW media. Three examples were studied: single-beam, counter-beam, and co-beam illumination. The results indicate that light scattering inside the solution does not produce stable SWWs. Next, we examined the formation of SWWs inside solid material layers for mis-aligned fibers. The self-writing process was indirectly monitored by observing the light emerging from the side of the material sample over time, and numerical simulations were carried out using the developed model. In the numerical predictions, a good qualitative agreement was observed with the experimental results. Therefore, the developed model can be used to more simply explore situations that would be difficult to implement experimentally. Several interesting applications have been identified, including Y-junctions and X-junctions, which facilitate a simpler alignment technique for the light and photopolymer media employed during the SWW process. Consequently, the solution can be used as a medium for interconnecting the different optical components in integrated photonic circuits or fiber optic applications.
